# Movement coaching: study protocol of a randomized controlled trial evaluating effects on physical activity and participation in low back pain patients

**DOI:** 10.1186/1471-2474-15-391

**Published:** 2014-11-22

**Authors:** Andrea Schaller, Ingo Froboese

**Affiliations:** Institute of Health Promotion and Clinical Movement Science, German Sport University Cologne, Am Sportpark Muengersdorf 6, 50933 Cologne, Germany; Center for Health through Sport and Movement, German Sport University Cologne, Am Sportpark Muengersdorf 6, 50933 Cologne, Germany

**Keywords:** Low back pain, Physical activity promotion, Inpatient rehabilitation, Multilevel approach, RCT

## Abstract

**Background:**

Chronic Low Back Pain is a complex syndrome with multifactorial bio-psycho-social etiology and interdependences. Thereby, physical activity seems to play an essential role regarding the prevention and rehabilitation of LBP. In consequence, physical activity and exercise therapy is an integral part of musculoskeletal rehabilitation in LBP. However, adherence to self-directed exercise and implementing a health-enhancing physical activity in daily routine after rehabilitation is a common problem for patients and only a few patients integrate health-enhancing physical activity and/or sport activities in their lifestyle. The present paper describes a comprehensive multilevel approach combining face-to-face intervention, telephone and internet aftercare *(Movement Coaching)*. Aim of the trial presented in this study protocol is to evaluate effectiveness of *Movement Coaching* compared to a control intervention.

**Methods/Design:**

The study is a prospective, single-blinded, monocenter randomized controlled trial (RCT) with three measuring points: T1 = start of inpatient rehabilitation; T2 = six months follow-up; T3 = twelve months follow-up. In total, 412 patients were recruited. The intervention involves small-group face-to-face contact during inpatient rehabilitation (two times, week 2 & 3) and telephone aftercare (week 8 & week 12 after rehabilitation) as well as internet-based aftercare (web 2.0 platform; available until six months after rehabilitation). Primary outcome is physical activity, assessed by GPAQ questionnaire. The final data collection is expected by April 2015.

**Discussion:**

Due to the burden of physical inactivity, there is a need to develop, evaluate and disseminate approaches that are effective in promoting physical activity and especially promoting the maintenance of physical activity in relevant target groups. Considering the high prevalence and socioeconomic impact of low back pain and its multifactorial etiology, low back pain patients seem to be a relevant target group for physical activity promotion. A multilevel approach to bridge the interface of (inpatient) rehabilitation and self-directed physical activity will help to target group-specific PA promotion.

**Trial registration:**

German Clinical Trials Register (DRKS)-ID: DRKS00004878.

## Background

Low Back Pain (LBP) has a high prevalence and a significant economic impact in Germany. Lifetime prevalence is estimated from 74% to 85% and almost 20% of the population are suffering either from severe or disabling back pain [1, 2]. In consequence, LBP is not only one of the leading causes of pain and disability but also a costly burden for the healthcare budget [3]. German healthcare costs related to LBP (ICD-10-GM: M45 – M54) were about 9 billion Euro in 2008, thereof 3,6 billion Euro for non-specific LBP (ICD-10-GM: M54). Thereby, costs mainly result from chronic LBP and related indirect costs, as LBP is one of the most frequent diagnoses causing work incapacity [4]. Overall, average costs of 1,322 Euro per LBP patient p.a. were calculated [2].

Chronic LBP is a complex syndrome with multifactorial bio-psycho-social etiology and interdependences [5, 6]. Besides physiological aspects [6], psychosocial aspects [7] and comorbidities (e.g. obesity), also sociodemographic aspects, such as educational level, are discussed to be a risk factor for LBP [1, 8, 9]. In addition to the aspects aforementioned, physical activity (PA) seems essential regarding the prevention and rehabilitation of LBP. LBP management guidelines point out the importance of PA in prevention as well as in management and rehabilitation of LBP [10–13]. Besides indication-specific aspects, regular PA with its manifold direct and indirect effects on health and wellbeing is considered an integral component of a healthy lifestyle [14]. World Health Organisation (WHO) guidelines recommend at least 150 minutes of moderate-intensity or 75 minutes of vigorous-intensity aerobic PA throughout the week or an equivalent combination of moderate- and vigorous-intensity activity. Independent of intensity, aerobic activity should be performed in bouts of at least 10 minutes duration [15]. These recommendations relate not only to sports, but also to other leisure activities, to work and transport activity. They address healthy adults and adults with chronic non-communicable conditions not related to mobility [15].

Based on the proven health-enhancing effects of PA on LBP and wellbeing, exercise therapy is an integral part of musculoskeletal rehabilitation in LBP patients [16] and therefore widely recommended as a successful therapy for that patient group [13, 17]. Nevertheless, only a few patients integrate health-enhancing physical activity and/or sport activities in their lifestyle. Based on the bio-psycho-social ICF model of functioning, disability and health [18], exercise therapy aims not only at improving body functions but also at improving participation by means of physiotherapy, therapeutic exercise, strengthening exercises, functional task-oriented training and aerobic training [19]. With regard to sustainability of exercise therapy in rehabilitation, its superior aim is to lead patients to an active lifestyle [12, 20]. Athough many studies have shown the effectiveness of exercise therapy in LBP patients, evidence on sustainability is lacking [21]. In consequence, adherence to self-directed exercise and implementing a health-enhancing PA in daily routine after rehabilitation is a common problem for patients [22]. Focussing PA behavior after rehabilitation, there only is evidence on short-term effects. In this context, barriers frequently mentioned are interface problems (e.g. the patients’ not knowing where to train) and lack of patient-oriented knowledge (e.g., being afraid of making mistakes by being physically active). In consequence, support in PA behavior at home is considered to be important [23].

This paper describes the *Movement Coaching* trial, evaluating a multilevel approach promoting PA. The primary research question of this study is:

- Does *Movement Coaching* lead to a higher level of PA in chronic LBP patients compared to a control group?

Related to the primary research question, the following hypotheses were developed:

1. The intervention group *(Movement Coaching)* shows 360 MET-min per week more PA than the control group at six months (resp. twelve months) after inpatient rehabilitation.
2. The intervention group *(Movement Coaching)* shows a higher level of leisure time PA than the control group at six months (resp. twelve months) after inpatient rehabilitation.
3. The intervention group *(Movement Coaching)* shows a higher level of transport PA than the control group at six months (resp. twelve months) after inpatient rehabilitation.

Additionally, the following secondary research question is evaluated by an explanatory approach:

- Is there a relationship between PA, subjective prognosis of employment and participation?

## Methods

### Study design

*Movement Coaching* is a prospective, single-blinded, monocenter randomized controlled trial (RCT) with three measuring points: T1 = start of inpatient rehabilitation; T2 = six months follow-up; T3 = twelve months follow-up. The study was approved by the German Sport University Cologne Ethics Committee (reference number: 56/12) and registered in the German Clinical Trials Register (ID: DRKS00004878). The *Movement Coaching* intervention will be compared with a control intervention at six (T2) and twelve (T3) months follow-up. The recruitment of patients began in Mai 2013 and was finished in April 2014. Six months follow-up (T2) will be completed in October 2014 and twelve months follow-up (T3) will be completed in April 2015. Reporting of the study will comply with the CONSORT statement for randomized trials [24, 25].

### Setting and participants

Participants were recruited from an inpatient medical rehabilitation center in North Rhine-Westphalia (Aggertalklinik). Eligible patients were invited to an informative meeting on the study as well as on health-enhancing effects of PA. The informative meeting was held during the first week of inpatient rehabilitation. Eligible patients had the possibility to participate in the study by giving informed consent form until the first unit of the intervention (three days). Eligibility criteria concerned: (1) age 18 to 65 years; (2) starting inpatient medical rehabilitation treatment because of LBP. Exclusion criteria included: (1) cognitive disorders; (2) lack of understanding the German language; (3) any kind of surgery within the last three months; (4) posttraumatic conditions (e.g., LBP after an accident); (5) a current state pension claim; (6) refusal of participating in the RCT. The study was conducted single blinded so that the patients did not know if they were randomized into the intervention group (*Movement Coaching)* or the control group.

### Study procedure

Eligible patients were randomly assigned to the intervention group *(Movement Coaching*) or the control group by means of a computer-generated random sequence table. Patients willing to participate in the study were screened on in- and exclusion criteria by a Movement-Coach. During the study period, both groups continued their medication and usual rehabilitation. All patients received usual inpatient rehabilitation. In this single-blind study, the Movement-Coach was not blinded since he/she conducted the intervention and the control intervention according to the randomization.

### Intervention

*Movement Coaching* is a multilevel approach and therefore comprises three different approaches: face-to-face contact (small group, three times during inpatient rehabilitation), telephone (week 8 & week 12 after rehabilitation) and internet (web 2.0 platform; available until six months after rehabilitation). Table 1 provides an overview of the objectives of the different approaches of *Movement Coaching* during the inpatient (face-to-face contact) and outpatient (telephone, internet) intervention period*.* The intervention is based on the “Rubicon Model of Action Phases” [26] and the “MoVo Process Modell” [27]. Additionally, contextual needs are considered within the concept of the intervention [28]. Concerning coaching methods and principles, the Movement-Coach does not give any rules, concrete suggestions or solutions on PA behavior to the patient. Instead, the Movement-Coach emphasizes the patient’s self-efficacy and individual resources to elaborate individual strategies on PA promotion [29].

**Table 1:** **Description of the movement coaching intervention**

Intervention measure	Main objectives
**Face-to-face contact**	
Movement Coaching I (inpatient; week 2)	Motivation
	Perceived consequences of PA behavior: Health-related risk perception
	Self-efficacy beliefs
	Planning individual physical activity after rehabilitation I
Movement Coaching II (inpatient; week 3)	Planning individual physical activity after rehabilitation II Self-efficacy beliefs
	Barriers and solution strategies
	Networking; places to be physically active at home
**Telephone aftercare**	
Telephone aftercare I (week 8 after inpatient rehabilitation)	Establishing a solid relationship of trust
	Current PA behavior of the patient
	Barriers and facilitators to transfer physical activity plans in daily living
	Further planning in PA activities
Telephone aftercare II (week 12 after inpatient rehabilitation)	Current PA behavior of the patient
	Barriers and facilitators to transfer physical activity plans in daily living
	Further planning in PA activities
**Internet based aftercare**	
Web 2.0 platform (until 12 months after inpatient rehabilitation)	Target group specific information on PA and LBP
	Communication platform

Face-to-face contact of *Movement Coaching* is conducted two times during the three week inpatient rehabilitation (week 2 & week 3). Maximum group size is eight persons and duration of the session is 60 minutes each.

Telephone aftercare comprises at least two calls of the Movement-Coach (week 8 & week 12 after rehabilitation). If the patient requests, further telephone coaching sessions are possible so that call frequency is flexible and can be tailored to the needs of the participant. Furthermore, patients can contact the Movement-Coach by telephone or internet. Main objective of telephone aftercare is to support the patient in his/her PA behaviour. If patients cannot be reached by telephone in the designated week, the Movement-Coach tries to contact him/her within the next two weeks. If a patient cannot be reached within the period of three weeks, the phone coaching is deleted without replacement. The telephone coaching is conducted on basis of a guideline and is scheduled for about 10 minutes. Duration may vary according to the individual needs of patients. All telephone coaching sessions are documented.

Additionally to telephone aftercare, patients have the possibility to use an interactive online platform until 12 months after inpatient rehabilitation. On this platform, patients receive further information on PA and LBP and have the possibility to communicate with the Movement-Coach or other patients.

Two Movement-Coaches were involved in conducting the intervention. Both have a Master’s degree in Sport Science with the main field of study in “Rehabilitation and Health Management”.

The control group receives two lectures on health-enhancing PA during inpatient rehabilitation, 30 minutes each. During the aftercare period, the patients have the possibility to download the lectures from a website. Contrary to the *Movement Coaching* intervention group, after inpatient rehabilitation no communication or interaction between coach and patient or the patients themselves is supported.

### Measurements

Data are collected by questionnaire at each of the three measurement points. The main outcome PA was operationalised by the Global Physical Activity Questionnaire (GPAQ) [30, 31]. GPAQ measures prevalence of physical inactivity as well as frequency per week and duration of PA differentiated in three domains, respectively life areas: work (paid and unpaid), transport (e.g., walking and cycling to get to and from places) and leisure time. For each domain, PA conducted for at least 10 minutes continuously during a usual week is assessed. Thereby, work and leisure time PA is measured intensity-specific, which allows to distinguish information on vigorous and moderate PA. Concurrent validity of GPAQ was assessed by comparison with International Physical Activity Questionnaire (IPAQ), a previously validated and accepted measure of physical activity. Thereby, GPAQ showed a moderate to strong positive relationship (concurrent validity: Spearman’s rho 0.45 – 0.65). Reliability was of moderate to substantial strength (kappa 0.67 to 0.73; Spearman’s rho 0.67 to 0.81) [31].

Additionally, a subgroup of 20 patients will receive an accelerometer for the measurement of objective PA at T2 (6 months follow-up). Objective PA will be measured through ActiGraph GT3X tri-axial accelerometers. Participants will be instructed to wear the monitor on a belt around their waist for seven days except during sleeping, showering or swimming. We offer financial incentives to complete questionnaires or to wear accelerometers. Table 2 gives a summary of all measures that will be collected.

**Table 2:** **Summary of measures**

	Instrument	Time of measurement^1^
**Primary outcome measure**		
Physical Activity	GPAQ [30, 31]	T1, T2, T3
	Actigraph	T2 (20 persons)
**Secondary outcome measures**		
Subjective prognosis of employment	SPE-Scale [32]	T1, T2, T3
Participation	IMET [33]	T1, T2, T3
**Person-related variables**		
Age, sex, height, weight,	Unstandardized questionnaire	T1
Education level	Unstandardized questionnaire	T1
Health Related Quality of Life	EQ-5D-5 L	T1, T2, T3
**Physical activity related variables**		
Barriers of PA	[34] (modified)	T2, T3
Perceived consequences of PA	[35]	T2, T3
Support of family and friends	Fuchs (modified)	T2, T3
**Indication-specific variables**		
Activities of daily living	FFbH-R [36]	T1, T2, T3
Pain	SF-36, NRS	T1, T2, T3
Disease duration	Unstandardized questionnaire	T1
Complications	Unstandardized questionnaire	T2, T3
Healthcare utilization	Unstandardized questionnaire	T2, T3
**Work-related variables**		
Period of sick listing	Unstandardized questionnaire	T1, T2, T3
Subjective workability	Unstandardized questionnaire	T1, T2, T3
Pension claim	Unstandardized questionnaire	T1, T2, T3

^1^T1 = start of inpatient rehabilitation; T2 = six months follow-up; T3 = twelve months follow-up.

### Sample size

The power calculation is based on the main hypothesis (hypothesis 1). Therefore it is assumed that the intervention group *(Movement Coaching)* shows 90 min per week more PA than the control group. The assumption is based on the estimation of a baseline PA level of 60 min per week in average. On the one hand, an increase of 90 min per week is considered realistic from a practical point of view, on the other hand it is considered a relevant effect as the achieved 150 min per week (assumption: baseline PA = 60 min per week + increase by Movement Coaching = 90 min per week) would go in line with the WHO recommendations on PA [15]. In this current RCT study, 264 participants (132 per group) were calculated to be necessary to detect the assumed relevant difference of 90 min moderate PA per week (~360 MET-min) between the two groups at a two-sided significance level of 0.05 (power 0.8). Anticipating on maximum loss to follow-up of 35% and using a non-parametric statistic test (+5%) the calculated target sample size is 370 patients.

### Statistical analysis

Descriptive statistics will be used to describe the main characteristics of the study population.

The primary analysis will be performed according to the per protocol principle. In addition, an intention-to-treat analysis will be performed. Differences between the intervention group (Movement Coaching) and the control group will be evaluated by Mann-Whithney-U Test at six month (T2) and twelve month follow-up. For all analyses, a two-tailed significance level of p <0.05 is considered to be statistically significant. All analyses will be carried out with IBM SPSS Statistics 21.0.

## Discussion

Low levels of PA in the population require treatment strategies in PA promotion. Therefore, there is a need to develop, evaluate and disseminate approaches that are effective in promoting PA and especially promoting the maintenance of PA. Furthermore, from a Public Health perspective, it is of utmost importance to reach a relevant target group for PA promotion.

Although the study is well-considered, several operational challenges have to be taken into account. One challenge is the recruitment of a sufficient number of patients. During the one-year recruitment, 412 patients could be included in the study. However, recruitment period had to be extended for three months to achieve the target sample size. Second, a selection bias needs to be controlled. Furthermore, the patients’ utilization of the interactive web 2.0 platform as well as target group specific development of the web content seems to be challenging. While planning the study, it is hard to anticipate how to design a motivating web 2.0 platform for CLBP patients after inpatient rehabilitation.

There are several strengths in the design of this study. First, the primary outcome measurement PA will not only be measured subjectively (questionnaires) but also objectively in a subgroup of patients. Second, as far as the authors know, the combination of face-to-face, telephone and internet intervention is new and user experiences of different stakeholders (e.g., patients, providers) can be used in order to improve each approach and/or improve the detailed concept of the comprehensive approach to reach the target group. Third, the 12-month follow-up will result in data about long-term effectiveness of PA promotion in CLBP patients. Furthermore, the examination of associations of PA, subjective prognosis of employment and participation is a promising approach to examine the relative significance of PA in regard of the rehabilitation’s primary objectives according to the ICF model.

CLBP patients seem to be a relevant target group for PA promotion. Based on the results of the current study, modification of Movement Coaching for further indications and target groups is planned.

## Abbreviations

PA: Physical activity
LBP: Low back pain
WHO: World Health Organization.
